# AMPK-Mediated Metabolic Switching Is High Effective for Phytochemical Levo-Tetrahydropalmatine (l-THP) to Reduce Hepatocellular Carcinoma Tumor Growth

**DOI:** 10.3390/metabo11120811

**Published:** 2021-11-29

**Authors:** Xunzhe Yin, Wenbo Li, Jiaxin Zhang, Wenjing Zhao, Huaxing Cai, Chi Zhang, Zuojia Liu, Yan Guo, Jin Wang

**Affiliations:** 1 School of Pharmacy, Changchun University of Chinese Medicine, Changchun 130117, China; xzyin@ciac.ac.cn (X.Y.); hxingcai@126.com (H.C.); baiyaoshi@126.com (C.Z.); 2 State Key Laboratory of Electroanalytical Chemistry, Changchun Institute of Applied Chemistry, Chinese Academy of Sciences, Changchun 130022, China; wbli@ciac.ac.cn (W.L.); jiaxin@ciac.ac.cn (J.Z.); xingyuewj@163.com (W.Z.); 3 Department of Chemistry and Physics, Stony Brook University, Stony Brook, NY 11794-3400, USA; jin.wang.1@stonybrook.edu

**Keywords:** AMPK, autophagy, cancer metabolism, hepatocellular carcinoma, levo-tetrahydropalmatine

## Abstract

Targeting cancer cell metabolism has been an attractive approach for cancer treatment. However, the role of metabolic alternation in cancer is still unknown whether it functions as a tumor promoter or suppressor. Applying the cancer gene-metabolism integrative network model, we predict adenosine monophosphate-activated protein kinase (AMPK) to function as a central hub of metabolic landscape switching in specific liver cancer subtypes. For the first time, we demonstrate that the phytochemical levo-tetrahydropalmatine (l-THP), a *Corydalis yanhusuo*-derived clinical drug, as an AMPK activator via autophagy-mediated metabolic switching could kill the hepatocellular carcinoma HepG2 cells. Mechanistically, l-THP promotes the autophagic response by activating the AMPK-mTOR-ULK1 and the ROS-JNK-ATG cascades and impairing the ERK/AKT signaling. All these processes ultimately synergize to induce the decreased mitochondrial oxidative phosphorylation (OXPHOS) and mitochondrial damage. Notably, silencing AMPK significantly inhibits the autophagic flux and recovers the decreased OXPHOS metabolism, which results in HepG2 resistance to l-THP treatment. More importantly, l-THP potently reduces the growth of xenograft HepG2 tumor in nude mice without affecting other organs. From this perspective, our findings support the conclusion that metabolic change is an alternative approach to influence the development of HCC.

## 1. Introduction

Hepatocellular carcinoma (HCC) is the sixth most common cancer, accounting for the fourth highest mortality rate worldwide [1]. Although the currently available clinical treatment methods, such as chemotherapy and radiation therapy, are greatly beneficial for the treatment of patients with HCC, the patients with 5-year survival rates are no more than 15% [2]. Therefore, it is necessary to develop more effective drugs for HCC. Phytochemical constituents are of particular importance as they help to improve the human health.

Autophagy is an intracellular evolutionarily conserved catabolic degradation process involving the proteins or damaged organelles [3]. These cytoplasmic macromolecules are delivered to lysosomes and then digested, ultimately producing basal buildings for cellular energy metabolism. This cellular self-digestion effectively maintains cellular homeostasis and mitigates metabolic stress under normal growth conditions. There is crosstalk between autophagy and apoptosis under physiological cellular stress conditions. Autophagy can control apoptosis and maintain cell homeostasis via alternative protective and non-protective autophagy in cancer [4]. The anti-proliferative effects of autophagy are attributed to the increased number of autophagosomes and the formation of autophagic vacuoles in cancer cells by increasing the conversion of the microtubule-associated protein light chain 3 (LC3)-I to LC3 II. The increased expression of LC3 II further initiates non-protective autophagy. A large number of phytochemicals exert anti-proliferative effects in different cancer types by inducing autophagy [5]. In many biological networks, autophagy plays important roles in providing the necessary biomass for cancer metabolism [6]. Enhancing autophagy significantly impairs mitochondrial metabolism and oxidative phosphorylation (OXPHOS). Inhibition of mitochondrial metabolism facilitates the generation of reactive oxygen species (ROS), whereas OXPHOS impairment leads to phosphorylation-related changes in the ROS-mediated extracellular signal-regulated kinase (ERK)/serine-threonine kinase (AKT) pathway. These alterations ultimately contribute to the initiation or progression of cancer [7]. Therefore, understanding the detailed mechanisms underlying such altered metabolism will be beneficial for cancer treatment [8].

Li and Wang developed a network model and demonstrated that the adenosine monophosphate-activated protein kinase (AMPK) is a primary regulatory element of energy metabolism, promoting metabolic switching to maintain energy homeostasis [9,10]. Here, in silico calculations revealed that AMPK is a key metabolic hub in the integrative network of cancer gene metabolism that regulates the metabolic landscape of HCC cells. We experimentally verified a new antitumor mechanism of levo-tetrahydropalmatine (l-THP), a *Corydalis yanhusuo*-derived bioactive alkaloid, in HepG2 cells by promoting autophagy via AMPK activation, leading to decreased metabolism due to ineffective OXPHOS. Additionally, l-THP stimulates mitochondrial ROS accumulation and facilitates the ERK/AKT signaling. These alterations sensitized HepG2 cells to apoptosis and inhibited the tumor growth in mice. Our findings suggest that l-THP may be used as an autophagy inducer for the treatment of HCC.

## 2. Results and Discussion

### 2.1. AMPK Is a Key Metabolic Hub in the Cancer Gene-Metabolism Integrative Network

**Figure 1:**
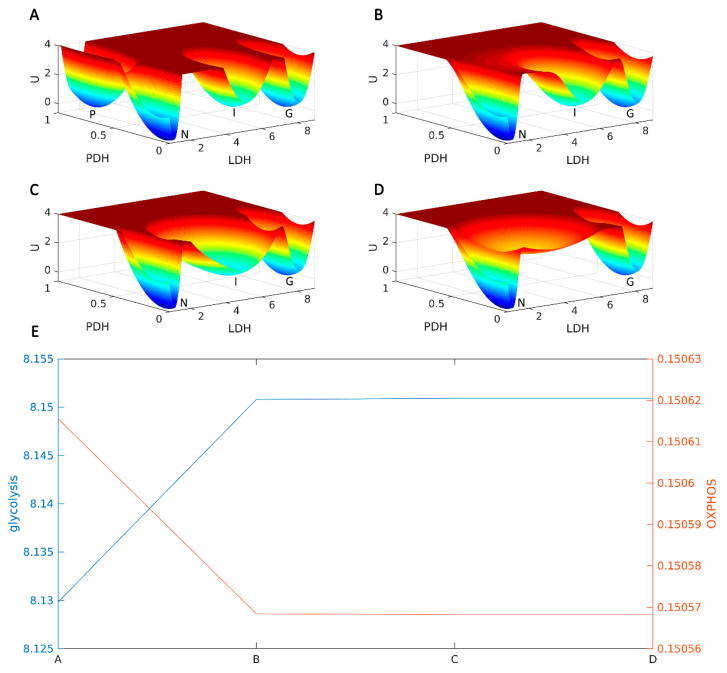
Metabolic landscape uncovers AMPK as a metabolic hub in cancer gene-metabolism integrative network. U is steady state probability distribution among variables. Lactate dehydrogenase (LDH) and pyruvate dehydrogenase (PDH) are chosen as an indicator of glycolysis and OXPHOS, respectively. Steady-state attractors, including normal state (*N*), cancer OXPHOS state (*P*), cancer glycolysis state (*G*), and cancer intermediate sate (*I*), are shown on the resulting quantified landscape topography. (**A**–**D**) Increasing AMPK expression results in a significant decline of OXPHOS level and a remarkable augment of glycolysis level. (**E**) The trend of AMPK-mediated metabolic switching in OXPHOS and glycolysis.

AMPK, as a central element of metabolism, regulates energy metabolism and coordinates the growth and metabolism in organisms [11,12]. Global quantification of the metabolic landscape is a promising strategy to clarify the potential mechanisms of cancer metabolism. We applied the cancer gene-metabolism integrative network model to uncover the underlying metabolic landscape regulated by AMPK activity. The majority of liver cancer subtypes rely on glycolysis to retain cancerous features [13]; therefore, we considered the cancer glycolysis state as the main metabolic phenotype in computational calculations. As shown in Figure 1, lactate dehydrogenase (LDH) and pyruvate dehydrogenase (PDH) are considered indicators of glycolysis and OXPHOS, respectively. Steady-state attractors, including normal state (*N*), cancer OXPHOS state (*P*), cancer glycolysis state (*G*), and cancer intermediate state (*I*), are shown on the resulting quantified landscape topography. The blue region corresponds to the low-potential area, whereas the red region corresponds to the high-potential area. When elevating the AMPK threshold value in the metabolic regulation network, the LDH level in the *G* state was much higher than that in the *N* state (Figure 1A–D). The *P* state rapidly disappears. Both *P* and *G* states can switch to each other through the *I* state. As AMPK is further elevated, the *I* state gradually disappears, but it remains trapped in the glycolytic attractor. The process of metabolic landscape switching resulted in the significant increase in the glycolysis levels, whereas the OXPHOS levels declined, as depicted in Figure 1E. The metabolic landscape provides a better guide for clarifying the adaptive mechanisms of AMPK involved in specific liver cancer subtypes.

### 2.2. l-THP Enhances Autophagy by Activating the AMPK-mTOR-ULK1 Axis

#### 2.2.1. l-THP Activates the AMPK-mTOR-ULK1 Signaling Pathway

**Figure 2:**
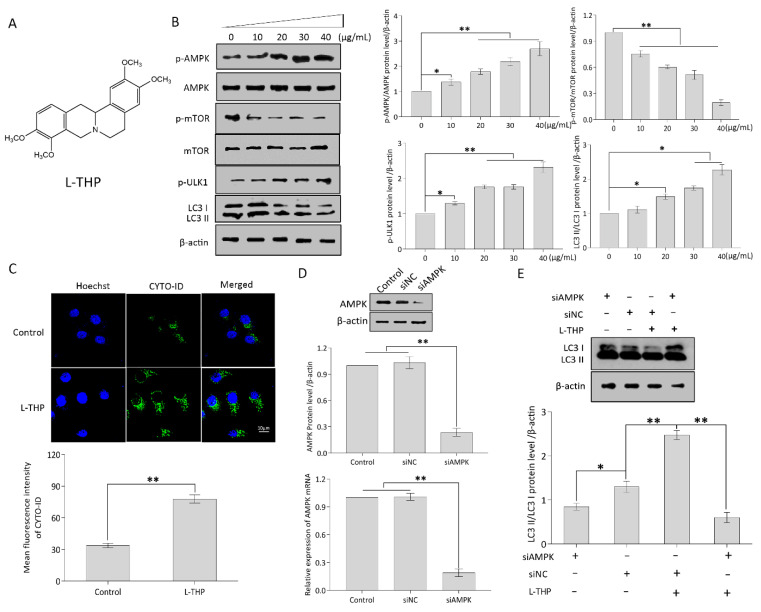
l-THP enhances autophagy through activating AMPK-mTOR-ULK1 cascade in HepG2 cells. (**A**) Chemical structure of l-THP. (**B**) Western blotting were performed by using the respective antibodies against LC3, p-ULK1, p-AMPK, AMPK, p-mTOR, mTOR, and β-actin. (**C**) After pretreatment with l-THP, the cells were stained with CYTO-ID and Hoechst 33,342 and confocal microscopy images were taken. (**D**) Knock-down AMPK of siRNA was presented by western blotting or quantitative RT-PCR analyses. (**E**) LC3 II/I ratio was measured after AMPK knockdown with siRNA. Confocal images were quantified by using Image J software. Data are presented as mean ± SD. * *p* < 0.05 and ** *p* < 0.01, versus the control, are significant difference.

l-THP (Figure 2A), a *Corydalis yanhusuo*-derived phytochemical component, exerts pharmacological effects, including anti-inflammatory and analgesic effects [14]. It also exerts anti-tumor effects on malignant tumors, such as melanoma, leukemia, and breast cancer via the p53-independent apoptotic pathway [15]. It is clinically used as an anxiolytic and sedative agent in China [16]. However, very little is known about its activity against HCC.

AMPK is a key energy sensor with a dual role in the organisms [17]. On the one hand, inactivating AMPK with pharmacological inhibitors can suppress mitochondrial OXPHOS to exert antitumor effect. For instance, combination of AMPK inhibitor Compound C and 2-deoxyglucose (2-DG) synergistically enhanced the cytotoxic potential in breast cancer cells [18]. Pan et al. reported that berberine directly induced apoptosis on drug-resistant breast cancer by AMPK downregulation [19]. On the other hand, AMPK activation can also act as a tumor suppressor. Mechanistically, active AMPK phosphorylates the ULK1 protein to initiate an autophagic cascade [20]. mTOR suppresses autophagy by inhibiting ULK1 phosphorylation [21]. Accordingly, when AMPK activation and mTOR inactivation occur simultaneously, this will produce a maximum activation of ULK1 and increase autophagy [22]. In HepG2 cells, the phosphorylated AMPK (p-AMPK)/AMPK ratio was significantly increased in a dose-dependent manner at the indicated concentrations of l-THP (Figure 2B). AMPK activation suggests that l-THP acts as an AMPK inducer, consistent with the mechanism of autophagy initiation [23]. Activation of AMPK inhibits the mTOR-dependent pathway, which plays a key role in autophagosome biogenesis [24]. To investigate whether l-THP regulates autophagy in HepG2 cells dependent on the mTOR pathway, p-mTOR and total mTOR proteins were examined. Compared to the vehicle control, a decline in p-mTOR/mTOR levels was observed in HepG2 cells treated with l-THP at the indicated concentrations (Figure 2C). As a direct target of mTOR, ULK1 activation represents a significant step toward autophagy induction. As shown in Figure 2B, ULK1 appears to be phosphorylated by l-THP as compared to the vehicle control. Additionally, LC3, a key regulator of autophagy, extensively participates in the entire process from autophagic vacuole formation to lysosomal degradation [25]. In particular, LC3 is considered a well-known marker of autophagosomes, and the high LC3 II/I ratio correlates with the autophagic activation due to the augmentation of autophagosomes. l-THP obviously increased the LC3 II/I ratio, causing a 2.2-fold increase at 40 µg/mL concentration compared to the vehicle control (Figure 2B). These results suggest that LC3 II accumulation is positively related to l-THP concentration.

#### 2.2.2. l-THP Enhances the Autophagic Flux in HepG2 Cells

Thereafter, autophagic flux was measured using CYTO-ID green autophagy dye to further verify the effect of l-THP on autophagy. The CYTO-ID probe serves as a specific marker that monitors the increment of autophagic compartments and is therefore associated with increased autophagic flux [26,27]. As a result, confocal images showed a 2.31-fold increase in the CYTO-ID fluorescence intensity in HepG2 cells treated with 40 µg/mL of l-THP compared with that of the control group (Figure 2C). To clarify the relationship between AMPK and autophagy, HepG2 cells treated with AMPK siRNA were cultured in media containing 40 µg/mL l-THP (Figure 2D). It could be clearly seen that knockdown of AMPK remarkably affected the protein expressions of LC3 II and LC3 I leading to autophagy recovery (Figure 2E), indicating that the enhancement of autophagy strongly depends on AMPK activity. Collectively, these results reveal that AMPK is an autophagic activator that promotes the AMPK-mTOR-ULK1 axis to adapt to l-THP stimulation.

### 2.3. l-THP Increases the Autophagic Flux via the ROS-JNK-ATG Cascade

#### 2.3.1. mtROS Molecular Pathway Is Essential to Autophagy Induction

**Figure 3:**
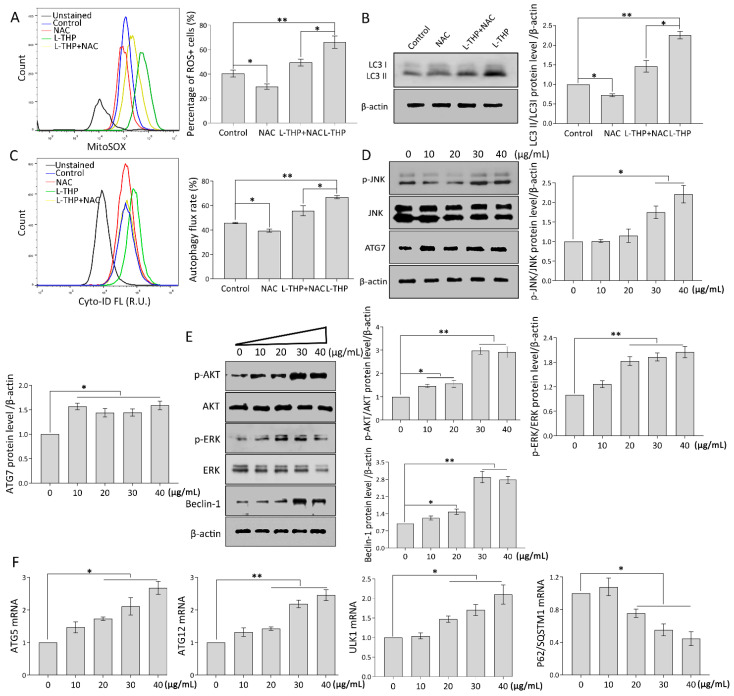
l-THP enhances autophagy flux via ROS-JNK-ATG axis. (**A**) l-THP induced mtROS accumulation in HepG2 cells treated with vehicle, 5 mM NAC, 40 µg/mL l-THP, or a combination of NAC and l-THP for 24 h. (**B**) LC3 level was evaluated by western blotting and (**C**) CYTO-ID dye signal was analyzed by flow cytometry. (**D**) p-JNK/JNK and ATG7 expression levels were evaluated. (**E**) p-ATK/AKT, p-ERK/ERK, and Beclin-1 were examined by western blotting. (**F**) mRNA expression of ULK1, ATG5, ATG12, and P62/SQSTM1 was detected using qPCR. Values are expressed as the mean ± SD of three independent experiments (*n* = 3). * *p* < 0.05 and ** *p* < 0.01 are considered to be statistically significant.

ROS have been proposed to have dual roles that can facilitate or delay the proliferation of cancer cells, depending on the cellular context and cell type [28]. Notably, abnormal mitochondrial ROS (mtROS) generation induces autophagy via the c-Jun N-terminal kinase (JNK)-ATG7 (autophagy-related gene 7) axis and ERK/PI3K cascade [29,30]. Thus, we further determined whether l-THP-induced autophagy was attributed to mtROS generation in HCC cells. As shown in the flow cytometric data (Figure 3A), l-THP significantly elevated mtROS overproduction, whereas NAC, a general ROS scavenger, suppressed the l-THP-induced mtROS accumulation in HepG2 cells. l-THP facilitates mtROS production, suggesting that enhanced autophagy increases the resilience of cells to mtROS. It is worth noting that the induced-l-THP LC3 II augmentation was significantly decreased in the presence of NAC (Figure 3B). This further resulted in the impairment of autophagic flux in HepG2 cells caused by the combination of l-THP and NAC (Figure 3C). In addition, emerging evidence has confirmed that the JNK/ATG7 signaling pathway participates in mtROS-mediated autophagy events [31]. Under conditions of high mtROS levels (40 µg/mL), we examined p-JNK and ATG7 expression levels. Interestingly, mtROS generation encouraged p-JNK/JNK expression in HepG2 cells, which led to ATG7 upregulation, and in turn, elevated autophagic flux (Figure 3D). mtROS-mediated autophagy has been observed in diverse cancer cells [32]. Autophagy is also involved in oncogenic and/or suppressive processes in tumors depending on the context and tumor stage, which makes it essential to be accurately evaluated in genetic models of tumors. The ERK/AKT cascade can initiate the autophagic process in various cancer cells [33,34]. Mechanistically, increased mtROS activates the ERK/AKT cascade, which further upregulates Beclin-1 levels and alternatively activates or inactivates the autophagic process. Considering the essential roles of both AKT and ERK pathways, we measured the phosphorylation levels of AKT and ERK. Notably, p-AKT/AKT and p-ERK/ERK levels were significantly increased with increasing l-THP concentration (Figure 3E). Similarly, Beclin-1, which subsequently participates in the formation of a large number of autophagic vacuoles, was positively upregulated by enhanced ERK levels (Figure 3E). Together, the increased mtROS facilitated the ERK/AKT cascade to promote autophagy initiation.

#### 2.3.2. Specific Genes Trigger Autophagy Occurrence

During autophagy, ATG12 and ATG5 gradually synthesize the ATG5-ATG12 complex that forms the regulatory system of the LC3 precursor and provides a preliminary basis for autophagy occurrence [35]. To assess whether autophagy induction was accompanied by specific gene modulation, qPCR analysis was performed in HepG2 cells treated with the indicated concentrations of l-THP. As shown in Figure 3F, mRNA expression of ATG12, ATG5, and ULK1 increased significantly in the presence of l-THP compared with that of the vehicle control. As a selective autophagy receptor, P62/SQSTM1 degrades ubiquitinated substrates when it interacts with LC3. Thus, the accumulation of P62/SQSTM1 mRNA levels was negatively correlated with autophagy activity. Of note, l-THP gradually decreased P62/SQSTM1 mRNA enrichment in HepG2 cells (Figure 3F), indicating that l-THP enhanced autophagy induction. Overall, these results reveal that mtROS production is closely related to the initiation of autophagy.

### 2.4. AMPK Activation by l-THP Decreased OXPHOS and Increased Glycolysis

#### 2.4.1. l-THP Changes the Metabolic Homeostasis between OXPHOS and Glycolysis

**Figure 4:**
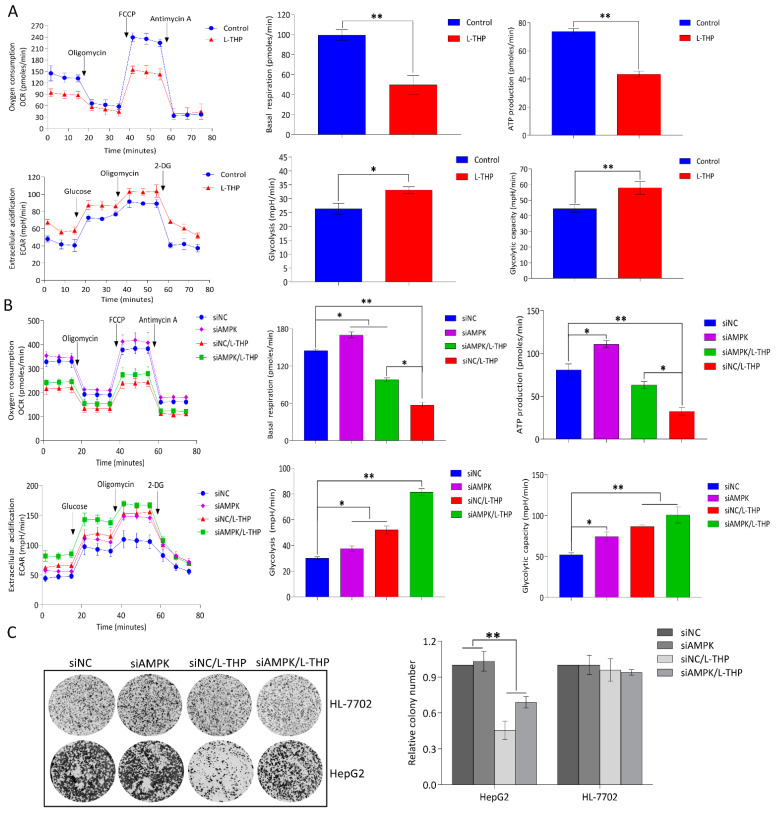
l-THP treatment leads to decreased OXPHOS and increased glycolysis in HepG2 cells. (**A**) The basal respiration level and ATP production rate in OCR as well as the glycolysis and glycolytic capacity in ECAR were examined using the Seahorse XFp analyzer. (**B**) OCR and ECAR were evaluated in HepG2 cells after silencing AMPK. (**C**) Colony formation assays were performed to assess the ability of l-THP to inhibit the growth of HepG2*^siAMPK+/−^* and HL-7702 cells. Quantitative data are expressed as the mean ± SD of three independent experiments (*n* = 3). * *p* < 0.05 and ** *p* < 0.01 are considered to be statistically significant compared to the control.

Autophagy can produce metabolic fuels that enable tumors with metabolic plasticity to thrive in an austere microenvironment [36]. Mitochondria as cellular “power engines” are important organelles in eukaryotic cells, which produce ATP via OXPHOS [37]. According to a previous report, energy status is pivotal for HCC maintenance [38]. Any metabolic pathway impairing the flexibility of energetic cancer cells affects tumor progression. Targeting anti-metabolism pathway may be exploited to treat cancer. As a successful example, Melatonin was found to withstand energy metabolism stress, which ultimately achieved the colorectal cancer therapy and inhibited lung cancer tumor growth by targeting GLUT3 metabolic pathway [39,40]. Utilizing the metabolic characteristics of tumor to find promising anti-metabolism pathway could open a new window for treating tumor. Thus, we investigated whether l-THP influences the balance between OXPHOS and glycolysis in HepG2 cells. The Seahorse XFp Extracellular Flux analyzer detected the metabolic flux of in HepG2 cells exposed to l-THP. Mitochondrial respiration was determined based on the oxygen consumption rate (OCR). Decreased basal OCR values were observed after 24-h of acute treatment with the indicated concentrations of l-THP (Figure 4A), indicating that the weakened OXPHOS caused l-THP toxicity. Simultaneously, ATP could also be attenuated to some extent after treatment with l-THP (Figure 4A), which provided evidence for decreased oxidative ATP production in l-THP-treated HCC cells. Collectively, the OCR data indicate that l-THP acutely damages mitochondrial function, losing the ability to obtain ATP from oxidative OXPHOS [41]. Glycolysis, the “Warburg effect,” is a hallmark of cancer [42]. Tumor cells have different metabolic changes and can adapt to changes in the metabolic environment through the conversion of glycolysis to OXPHOS [43]. Notably, the opposite trend was observed for glycolysis, as indicated by the extracellular acidification rate (ECAR) in HepG2 cells treated with l-THP (Figure 4A). Taken together, the OCR and ECAR results indicated that acute treatment with l-THP had an immediate but reversed effect on glycolysis in addition to decreasing the ability of HepG2 cells to induce mitochondrial respiration.

#### 2.4.2. AMPK Is a Hub for Metabolic Switching and Proliferation in HepG2 Cells

Pharmacological strategies that selectively target metabolic reprogramming have become progressively promising. The metabolic landscape shown in Figure 1 suggests an important role of AMPK, as a metabolic hub, between OXPHOS and glycolysis energy switching in the cancer gene-metabolism network. To experimentally elucidate the role of AMPK in regulating metabolism, HepG2 cells transfected with AMPK siRNAs were treated with l-THP. The basal OCR and ATP production rate of HepG2*^siAMPK+^* cells was significantly higher than that of HepG2*^siAMPK−^* cells treated with the same concentrations of l-THP, meanwhile the glycolysis rate and glycolysis capacity were increased in HepG2*^siAMPK+^* cells compared to that in HepG2*^siAMPK−^* cells (Figure 4B). This work provides a solid basis to better understand the role of AMPK in metabolic switching.

Autophagy has a “cytotoxic or inhibitory function” that can lead to autophagic cell death. In addition, metabolic reprogramming caused by mitochondrial dysfunction has been shown to inhibit tumor growth in vivo [44]. HepG2 cell colony formation was markedly inhibited in the presence of l-THP. In contrast, l-THP showed much less inhibition of colony formation after AMPK knockdown in HepG2 cells (Figure 4C). The results suggested that increase in OXPHOS and glycolysis by silencing AMPK had a significant resistance to the l-THP treatment. Notably, an xCELLigence real-time cellular analysis showed that l-THP suppressed HepG2 cell proliferation in a time-dependent manner via the mitochondrial apoptosis pathway and cell-cycle rearrangement (Supplementary Figures S1–S4). These observations strongly support the conclusion that inhibition of cell proliferation and growth caused by l-THP largely depended on the AMPK-mediated OXPHOS metabolic pathway. Thereafter, we determined the cytotoxicity of l-THP in human normal liver HL-7702 cells to assess its specificity towards cancerous cells. Compared to HCC cells, l-THP exhibited a slight inhibition of HL-7702 cells at a concentration of l-THP, which is toxic to cancer cells (Figure 4C). The results clearly showed that l-THP specifically inhibited HCC cell proliferation without obvious cytotoxicity towards normal cells.

### 2.5. l-THP Inhibits Tumor Growth in the Nude Mice Model

**Figure 5:**
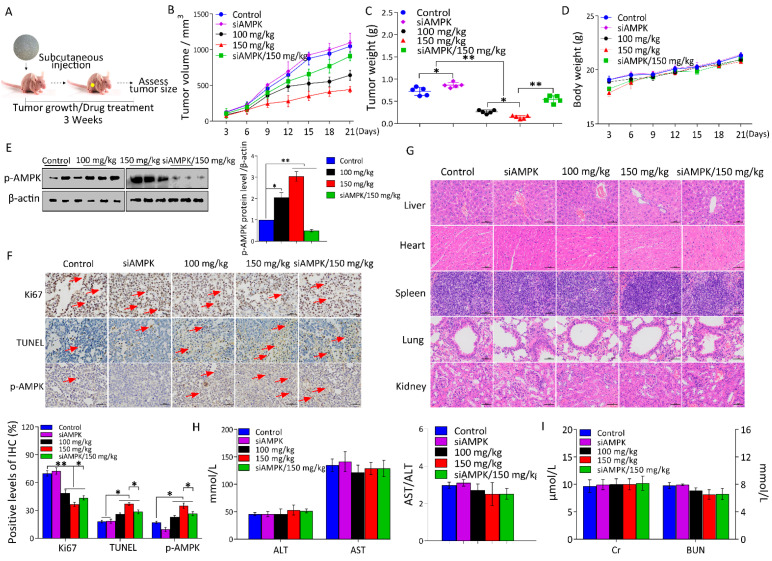
l-THP reduces the liver tumor growth by activating AMPK in vivo. (**A**) Flow chart of l-THP treated-BALB/c nude mice. (**B**–**D**) The tumor volumes, tumor weights, and body weights of the nude mice treated with or without l-THP were analyzed. (**E**) Western blotting using the lysates of isolated tumors and the indicated antibodies. (**F**) The expression levels of Ki67 and p-AMPK in the tumor tissues were detected by IHC and apoptosis was measured by TUNEL staining. Scale bar = 50 μm. (**G**) H&E staining was used to observe the histomorphology of the liver, heart, spleen, lung, and kidney tissues. Scale bar = 50 μm. (**H**,**I**) The serum levels of ALT, AST, Cr, and BUN in the mice from each group were detected. Scale bar equals to 100 μm. * *p* < 0.05 and ** *p* < 0.01 are considered to be statistically significant compared to the control.

In accordance with the effect of l-THP on the cell proliferation in vitro, we next evaluated the antitumor effect of l-THP on tumor growth in a xenograft model of liver cancer. Under energetic stress, activating AMPK suppresses the cell growth and proliferation, indicating that AMPK is associated with the tumor suppressor pathway [45]. To validate this, we constructed the HepG2 or HepG2*^siAMPK+^* xenograft model. Mice were injected subcutaneously with both the cells to provide the genetic evidence that AMPK can display tumor suppressor activity in vivo (Figure 5A). Results demonstrated that l-THP significantly suppressed the tumor growth, as reflected by a decrease in tumor volume (Figure 5B). In contrast, mice of the siAMPK group exhibited increased tumor volume compared to the control group. The average tumor weights of the l-THP treatment group of 100 mg/kg and 150 mg/kg were 63.7% and 80.1% lower than those of the control group, respectively (Figure 5C). In contrast, the AMPK knockdown cell strains (siAMPK/150 mg/kg) exhibited tumors with increased weight compared to those of the l-THP treatment group (150 mg/kg) (Figure 5C), suggesting that AMPK activity leads to a key genetic event that results in tumorigenesis [45]. However, the mice injected with l-THP did not exhibit any body weight loss or apparent toxicity (Figure 5D). The data suggest that AMPK is essential for the maintenance of liver tumors. Based on these observations, we performed IHC staining and western blotting to analyze the p-AMPK levels of tumor sections compared to that of the control mice. We found that l-THP remarkably increased the p-AMPK levels in the xenograft tumor sections (Figure 5E,F). Moreover, IHC staining results demonstrated that the l-THP treatment displayed lower cell proliferation indices (Ki67-positive) compared to the control tumors in xenograft tumor tissues (Figure 5F). Similarly, the terminal deoxyribonucleotidyl transferase (TDT)-mediated dUTP nick-end labeling (TUNEL) assay displayed a prominent augment of TUNEL-positive cells (apoptosis) in the l-THP-treated tumors (Figure 5F). H&E staining showed that l-THP administration did not cause changes in the liver, heart, spleen, lung, and kidney tissues of the model mice (Figure 5G). The serum levels of AST, ALT, Cr, and BUN showed little significant changes compared to the control group (Figure 5H,I), which indicated that the l-THP treatment did not cause any pathological damage or toxicity in the organs. These findings suggest that AMPK activation opposes tumor development, which is indeed a tumor suppressing pathway that restrains the tumor growth. Together, these findings indicate that l-THP may be used to selectively upregulate AMPK to suppress the liver tumor growth in vivo.

## 3. Conclusions

**Figure 6:**
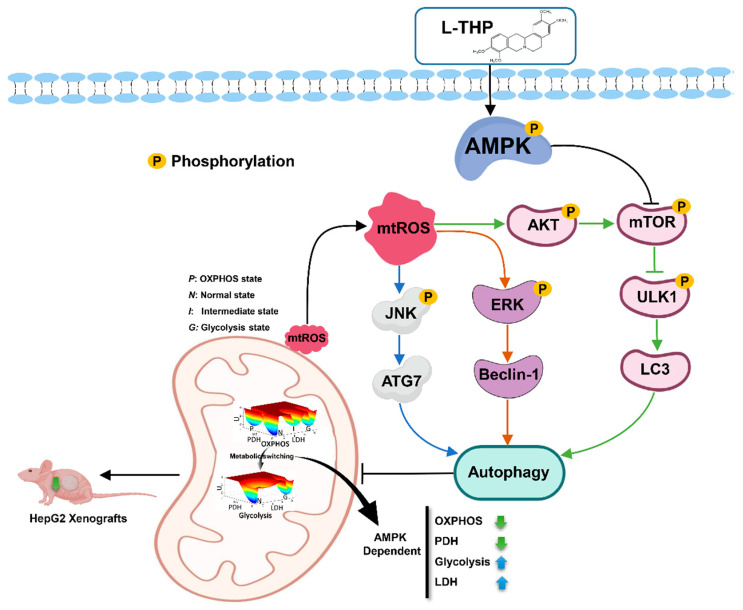
AMPK-mediated metabolic switching is high effective for l-THP to reduce HCC tumor growth in mice.

In this work, we uncovered a novel mechanism underlying the antitumor activity of the phytochemical, l-THP, which promotes autophagy via multiple signaling pathways, including the AMPK-mTOR-ULK1 cascade, ROS-JNK-ATG axis, and the ERK/AKT signaling pathway. Further, l-THP sensitizes mitochondrial damage-dependent HCC cell death, which finally leads to the decreased metabolism due to ineffective OXPHOS (Figure 6). Therefore, the results of our study indicate that l-THP is a natural autophagy inducer, which may be used as a potential therapeutic agent for HCC treatment.

## 4. Materials and Methods

### 4.1. Materials and Reagents

l-THP (ST8100, purity ≥ 98%) was purchased from the National Institutes for Food and Drug Control (NIFDC, Beijing, China). Dulbecco’s modified Eagle medium (DMEM), fetal bovine serum (FBS), streptomycin and penicillin were purchased from Gibco (Waltham, MA, USA). Bovine serum albumin (BSA) and *N*-acetylcysteine (NAC) were obtained from Sigma-Aldrich (St. Louis, MO, USA). Antibodies against LC3 (#ab192890), p-AMPK (#ab92701), AMPK (#ab32047), p-mammalian target of rapamycin (mTOR) (#ab109268), mTOR (#ab109268), p-unc 51-like autophagy activating kinase 1 (ULK1) (#ab133747), Beclin-1 (#ab210498), p-AKT (#ab38449), AKT (#ab81283), p-c-Jun N-terminal kinase (JNK) (#ab124956), JNK (#ab208035), autophagy-related (ATG)-7 (#ab52472), p-ERK (#ab201015), ERK (#ab184699), and β-actin (#ab6276) were supplied by Abcam (Cambridge, UK). The fluorescein isothiocyanate (FITC) Annexin V apoptosis detection kit, PI/RNase staining buffer, anti-caspase-9 (#551247), anti-cytochrome *c* (#556433), and anti-caspase-3 (#610323) antibodies were purchased from BD Biosciences (New York, NY, USA). Anti-mouse or anti-rabbit horseradish peroxidase (HRP)-conjugated secondary antibodies (#ZB-2305, #ZB-2301, respectively) were purchased from ZSGB-Bio (Beijing, China). A chemiluminescent HRP substrate was purchased from Millipore (Billerica, MA, USA). Crystal violet staining solution was obtained from Beyotime Biotechnology (Shanghai, China). MitoSOX™ Red mitochondrial superoxide indicator was purchased from Invitrogen (Waltham, MA, USA). CYTO-ID and Hoechst 33,342 were supplied by Enzo Life Sciences (New York, NY, USA) and specific small interfering RNAs (siRNAs) were purchased from Ribo-Bio (Guangzhou, China). UNIQ-10 column trizol total RNA isolation kit was obtained from Sangon Biotech (Shanghai, China), and the RNA reverse transcription kit and SYBR qPCR Master Mix were purchased from Promega (Madison, WI, USA). Primers were synthesized by Comatebio (Jilin, China). Mito stress and glycolysis stress test kits were supplied by Agilent Technologies (Santa Clara, CA, USA).

### 4.2. Cell Culture

The HCC cell line, HepG2, and human immortalized hepatocyte cell line, HL-7702, were obtained from the Chinese Academy of Science Type Culture Collection (Shanghai, China). HepG2 cells were cultured in DMEM with 10% (*v/v*) FBS supplemented with 1% streptomycin-penicillin antibiotics and HL-7702 cells were cultured in the Roswell Park Memorial Institute (RPMI)-1640 medium with 20% (*v/v*) FBS supplemented with 1% antibiotics, respectively, in a humidified atmosphere of 5% carbon dioxide (CO_2_) at 37 °C.

### 4.3. Self-Consistent Mean Field Approximation Approach

In this study, we applied the Self-Consistent Mean Field Approximation approach for an individual variable [9]. Briefly, the Gaussian distribution ansatz was used as an approximation to calculate the probability. The mean and variable are necessary to compute the probability. In the system with fluctuating environments, the dynamics can be described by the stochastic ordinary differential equations as $\dot{x}=F\left(x\right)+\zeta$. Here, x(t) represents the vector of the gene expression and enzyme concentration levels and F(x) represents the vector for the driving force between each regulation or interaction. Extrinsic and intrinsic fluctuations are important for the biological systems. The fluctuations term is added to the force as $\dot{x}=F\left(x\right)$, the deterministic dynamics of the system. The fluctuation term ζ is assumed to follow Gaussian distribution and the correlation functions are given as: $\zeta_{j}\left(x,t\geq 0\right)$ and $\langle \xi_{i}\left(x,t\right)\zeta_{j}\left(x,\;t\prime \right)\rangle =2D_{\mathrm{ij}}\delta_{\mathrm{ij}}\delta \left(t-t\prime \right)$, where $\delta_{\mathrm{ij}}=1$ for i = j and δij=0 for i ≠ j. Here δ(t) is the Dirac delta function and D is diffusion coefficient matrix. The fluctuation term is associated with the intensity of cellular fluctuations resulting from external environmental or internal environmental sources.

### 4.4. Detection of Autophagy Using Confocal Microscopy

HepG2 cells were seeded into a glass bottom culture dish (NEST Biotech, Wuxi, China) at a density of 5 × 10^4^ cells/well. For imaging studies, the cells were cultured in a chamber under incubator conditions (10% DMEM at 37 °C, 5% CO_2_). After l-THP (40 μg/mL) treatment, the overlay of CYTO-ID- and Hoechst 33342-stained cells was used to identify autophagosomes. Images were captured and processed by a Nikon confocal laser scanning (Tokyo, Japan).

### 4.5. Detection of Mitochondrial ROS Production by MitoSOX

In the live-cell mitochondria, MitoSOX Red reagent is oxidized by superoxide and exhibits red fluorescence. The level of mitochondrial ROS (mtROS) in cells was measured by MitoSOX fluorescence reagent using a flow cytometer. After 24 h of the treatment, with or without NAC (5 mM), the indicated concentration of l-THP was added into the wells. The cells (1 × 10^6^ to 1 × 10^8^ cells/mL) were trypsinized and incubated with the MitoSOX fluorescence reagent (5 μM) at 37 °C in the dark for 10 min. Thereafter, the cells were washed twice with warm buffer. The intensity of cell fluorescence was analyzed by an Accuri C6 Flow Cytometer (Franklin Lakes, NJ, USA). MitoSOX fluorescence was measured at 561 nm.

### 4.6. Autophagic Flux Analysis

An autophagic flux expression vector of HepG2 cells was used to detect and quantify the l-THP-induced autophagic cells using a CYTO-ID detection reagent. Cells were maintained as per the standard tissue culture practice and cultured overnight to let them enter into log phase in a humidified incubator at 37 °C and 5% CO_2_, followed by incubation with or without l-THP (40 μg/mL) and NAC (5 mM). After 24 h incubation, the treated HepG2 cells were detached using trypsin and resuspended in 1 × assay buffer or phenol red-free culture medium containing 5% FBS. Then, the cancer cells were stained with the CYTO-ID stain solution at 37 °C for 30 min. Thereafter, the cells were washed and resuspended in 1 mL of 1 × assay buffer. The samples were analyzed using an Accuri C6 flow cytometer and the quantified using the FlowJo v.10 software (BD Biosciences, Franklin Lakes, NJ, USA).

### 4.7. Western Blotting Analysis

Proteins (30 µg/well) were separated by sodium dodecyl sulfate-polyacrylamide gel electrophoresis (SDS-PAGE) and transferred to a polyvinylidene fluoride (PVDF) membrane (Millipore, Billerica, MA, USA). Membranes were blocked with skim milk and incubated overnight with a monoclonal primary antibody. The membranes were washed three times with the tris-buffered saline with tween (TBST) and incubated with the respective secondary antibodies (1:2000). The protein bands were visualized by Bio-Imaging Systems (DNR, Jerusalem, Israel) using a chemiluminescent HRP substrate. All quantifications of protein expression were made using ImageJ180 software (Bethesda, MD, USA) and normalized to β-actin.

### 4.8. siRNA Transfection

siRNAs were transfected using the riboFECT CP transfection. Thereafter, HepG2 cells were lysed and the expression levels of AMPK were assayed by western blotting.

### 4.9. RNA Extraction and Quantitative Reverse Transcription-Polymerase Chain Reaction (qRT-PCR) Analysis

The total RNA from l-THP-treated HepG2 cells was isolated using the UNIQ-10 column trizol total RNA isolation kit. A complementary cDNA synthesis kit was used for reverse transcription of RNA. Real-time qPCR was performed using the SYBRGoTaq qPCR master mix and the reaction was run on the ABI 7500 detection system. The PCR primers used are listed in Table S1. The mRNA expression was computed using the formula 2^−ΔΔCt^, where ΔΔCt = ΔCt (target gene) − ΔCt (β-actin gene), which was presented as the fold change in gene expression normalized to β-actin. The Minimum Information for Publication of Quantitative Real-Time PCR Experiments (MIQE) guidelines were followed for the qRT-PCR experiments.

### 4.10. Colony Formation Assay

HepG2 and HL-7702 cells were plated in 6-well culture plates (NEST Biotech, Wuxi, China) at 3000 cells/well. After approximately 2 weeks, the cell colonies fixed in paraformaldehyde (Solarbio, Beijing, China) were stained with crystal violet staining solution and photographed. Finally, the colonies were dissolved in dimethyl sulfoxide and the absorbance was measured at 570 nm using a NanoQuant autoreader (Tecan, Mannedorf, Switzerland).

### 4.11. Cell Metabolism Measurements

First, 5 × 10^3^ cells/well were seeded in XFp cell culture microplates a day before performing the assay. The oxygen consumption rate (OCR) and extracellular acidification rate (ECAR) were all measured using the Agilent’s Seahorse Bioscience XFp Extracellular Flux Analyzer (Santa Clara, CA, USA) according to the manufacturer’s instructions. Experiments were conducted in the XF assay medium (pH 7.3–7.4) containing 2.5 M glucose, 0.2 M glutamine, and 0.1 M sodium pyruvate. Then, 1 µM oligomycin, 1 µM FCCP, and 0.5 µM rotenone were used to measure the cellular mitochondrial function. On the other hand, 0.2 M glutamine, 10 mM glucose, 10 µM oligomycin, and 50 mM 2-deoxy-glucose (2-DG) were used to measure glycolytic capacity. ECAR and OCR were generated using the Wave Desktop v.2.4 software (Agilent, Santa Clara, CA, USA).

### 4.12. Xenograft Tumor Model BALB/c Nude Mice and Treatment Strategies

Female BALB/c nude mice (6-weeks old) were obtained from the Beijing Vital River Laboratory Animal Technology Co., Ltd. (Beijing, China) for in vivo experiments. All experimental procedures were approved by the Animal Care and Use Committee of Wish Detection Technology (Approval Number: SYXK 2019-0007, Changchun, China) and performed in strict accordance with legislation regarding the use and care of laboratory animals in China. The xenograft models were established by subcutaneous injection of HepG2 and/or HepG2*^siAMPK+^* cells (2 × 10^6^ cells/200 µL) into the side of the rib. The mice were randomly divided into five groups (*n* = 5/group): Group 1 (HepG2 cells), Group 2 (HepG2*^siAMPK+^* cells), Group 3 (HepG2 cells, 100 mg/kg of l-THP via oral gavage), Group 4 (HepG2 cells, 150 mg/kg of l-THP via oral gavage), and Group 5 (HepG2*^siAMPK+^* cells, 150 mg/kg of l-THP via oral gavage). Oral gavage was administered daily for 3 weeks. The length and width of the xenograft tumors were measured before the end of the experimental period. Mice were anesthetized using ether (#10009318; Sinopharm, Beijing, China), and all mice were sacrificed. Blood samples were collected for the analysis of aspartate aminotransferase (AST), alanine aminotransferase (ALT), creatinine (Cr), and blood urea nitrogen (BUN) levels. The xenografted tumors were removed and weighed, and other tissues were isolated and harvested for hematoxylin-eosin staining (H&E), immunocytochemistry (IHC), or western blotting assays.

### 4.13. Statistical Analysis

GraphPad Prism Software v.7.04 (GraphPad, San Diego, CA, USA) was used for the statistical evaluation of experimental data. All experimental data are presented as the mean ± standard deviation (SD), and all analyses were performed in triplicate. Statistical comparisons between two groups were performed by unpaired Student’s *t*-test, and comparisons between multiple groups were assessed by one-way analysis of variance (ANOVA). Differences were considered statistically significant at * *p <* 0.05 and ** *p <* 0.01.

## Data Availability

The data presented in this study are available in article.

## Supplementary Materials

The following are available online at https://www.mdpi.com/article/10.3390/metabo11120811/s1, Table S1: Primers used for real-time PCR analysis, Figure S1: Effect of l-THP on HepG2 cell proliferation was independently evaluated using the real time cellular assay xCELLigence system, Figure S2: Relative density of caspase-3, caspase-9, and cytochrome *c* proteins was quantitated and plotted, respectively, Figure S3: Representative images of cell populations in G1, G2, and S phases were analyzed by flow cytometry in HepG2 cells, Figure S4: Apoptotic cells were analyzed by flow cytometry following Annexin V-FITC/PI staining.
