# Supplementary material for: AMPK-Mediated Metabolic Switching Is High Effective for Phytochemical Levo-Tetrahydropalmatine (l-THP) to Reduce Hepatocellular Carcinoma Tumor Growth
Source: Metabolites. 2021 Nov 29;11(12):811. doi: 10.3390/metabo11120811 (PMC8703446; doi:10.3390/metabo11120811)
Supplement: Supplementary file 1 [file metabolites-11-00811-s001.zip › metabolites-1470107-supplementary.pdf]

Supplementary Materials

AMPK-Mediated Metabolic Switching Is High Effective for Phytochemical Levo-Tetrahydropalmatine (L-THP) to Reduce Hepatocellular Carcinoma Tumor Growth

This file includes:
S1 Materials and Methods
Table S1
Figure S1, S2, S3, S4

S1. Materials and Methods

1.1. Materials

FITC Annexin V apoptosis detection kit, PI/RNase staining solution, anti-caspase-9, anti-cytochrome *c*, and anti-caspase-3 antibody were purchased from BD Biosciences (New Jersey, USA).

1.2. Continuous cell monitoring with the xCELLigence system

For continuous monitoring of changes in cell growth, cells (5 × 10^3 cells/well) were seeded onto E-plates, and then the E-plates were placed onto the xCELLigence system (ACEA Biosciences, CA, USA). Cells were grown overnight, and impedance was measured every 15 min, prior to treatments. The principle of the xCELLigence system is that cells grown on the surface of electrodes produce an increase in impedance value whose magnitude is determined by the number of cells and the degree of cell adhesion. Cell proliferation was continuously monitored throughout the experiments by measuring changes in the electrode/solution interface.

1.3. Western blotting analysis

Equal amounts of protein (30 μg) were separated on 10% acrylamide sodium dodecyl sulfate polyacrylamide gel electrophoresis (SDS/PAGE) and transferred to PVDF membranes. Membranes were blocked with 5% skim milk for 2 h at room temperature, and incubated with monoclonal primary antibody at 4 °C overnight. The membranes were washed three times with 5% tween-20 in Tris-HCl buffered saline, incubated with anti-mouse or anti-rabbit HRP-conjugated secondary antibodies (1:2000) for 1 h at room temperature. The protein band signals were detected by a Bio-Imaging Systems using a

Chemiluminescent HRP Substrate. All the quantifications of protein expression were made using the Image J software and normalized with β-actin.

1.4. Annexin V assay

HepG2 cells were seeded at a density of 200,000 cells/well in a 6-well plate and cultured for 24 h. After L-THP 24h-treatment, an Annexin V-FITC assay was conducted following the manufacturer's instructions. Annexin V-FITC binding was detected by flow cytometry acquiring a minimum of 20,000 events from each sample. An apoptotic data analysis was performed using FlowJo Software v10 (BD Biosciences, New Jersey, USA).

1.5. Cell cycle distribution analysis

After L-THP treatment, the cells were washed with PBS (pH 7.4) before being fixed in 75% ethanol overnight at 4 °C. Subsequently, the cells were centrifuged and the residual alcohol was aspirated; cells were washed with PBS, resuspended and stained with PI/RNase staining solution, after that incubated at room temperature in the dark for 30 min. Finally, 20,000 events were evaluated in each sample by Accuri C6 flow cytometer. Analysis of data was used the ModFit LT software (Verity Software House, USA).

Supplementary Table

Table S1. Primers used for real-time PCR analysis.

Gene	Orientation	Sequence (5′ to 3′)
β-actin	Forward	GGAGCGAGATCCCTCCAAAAT
	Reverse	GGCTGTTGTCATACTTCTCATGG
p62/SQSTM1	Forward	GCACCCCAATGTGATCTGC
	Reverse	CGCTACACAAGTCGTAGTCTGG
ATG5	Forward	AAAGATGTGCTTCGAGATGTGT
	Reverse	CACTTTGTCAGTTACCAACGTCA
ATG12	Forward	CTGCTGGCGACACCAAGAAA
	Reverse	CGTGTTCGCTCTACTGCCC
ULK1	Forward	GGCAAGTTCGAGTTCTCCCG
	Reverse	CGACCTCCAAATCGTGCTTCT

Supplementary Figures and Legends

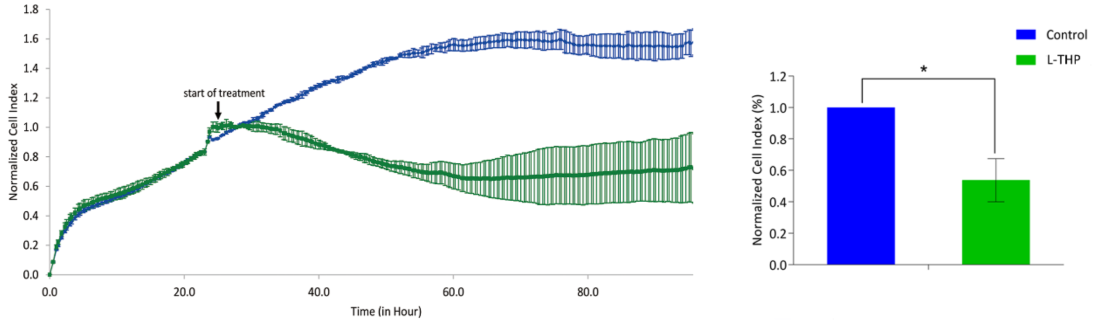

Figure S1. Effect of L-THP on HepG2 cell proliferation was independently evaluated using the real time cellular assay xCELLigence system. The data shown represent the mean ± SD, differences at *$P < 0.05$ and **$P < 0.01$were considered statistically significant.

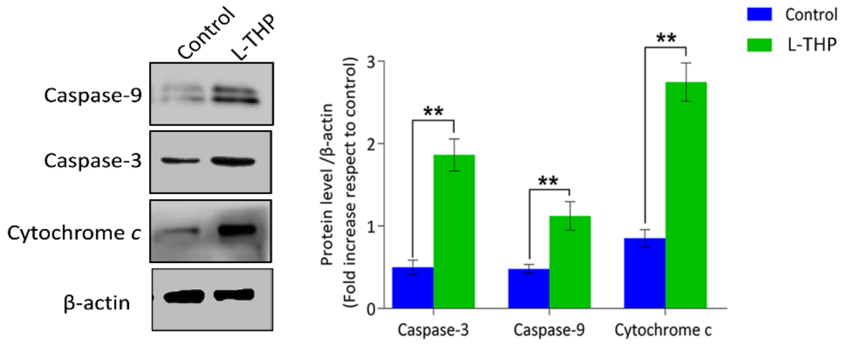

Figure S2. Relative density of caspase-3, caspase-9, and cytochrome *c* proteins was quantitated and plotted, respectively. The data shown represent the mean ± SD, differences at *$P < 0.05$ and **$P < 0.01$ were considered statistically significant.

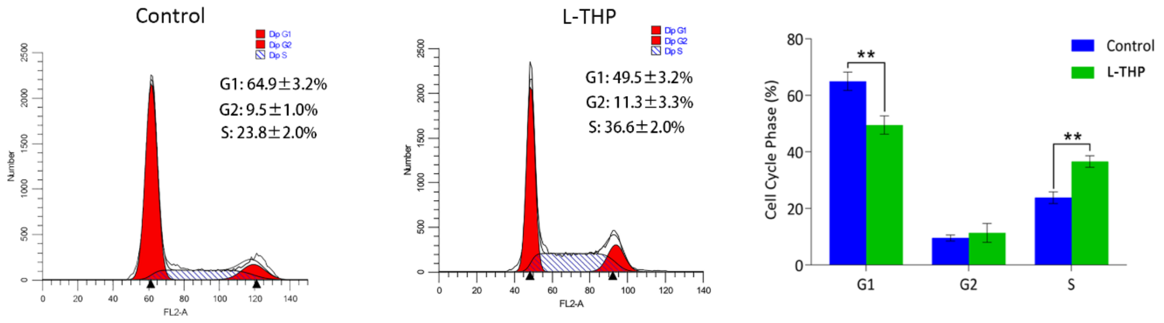

Figure S3. Representative images of cell populations in G1, G2, and S phases were analyzed by flow cytometry in HepG2 cells. The data shown represent the mean ± SD, differences at *P < 0.05 and **P < 0.01 were considered statistically significant.

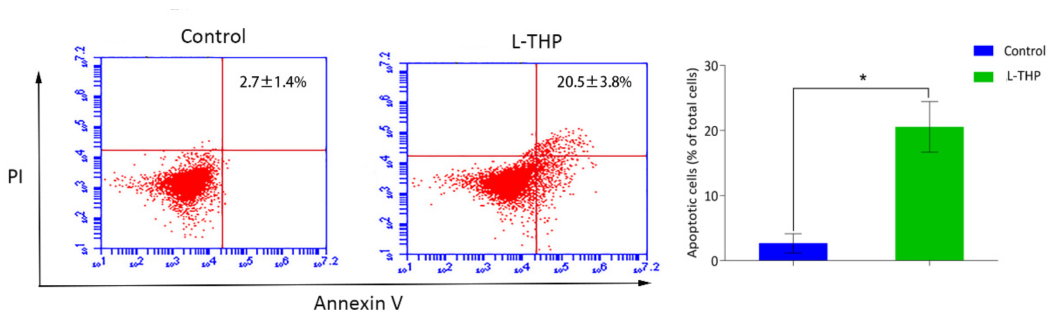

Figure S4. Apoptotic cells were analyzed by flow cytometry following Annexin V-FITC/PI staining. The data shown represent the mean ± SD, differences at *P < 0.05 and **P < 0.01 were considered statistically significant.
